# Ultrasonographic evaluation of suprahyoid muscle thickness during the transition to full oral feeding in preterm infants

**DOI:** 10.1007/s00431-026-07019-w

**Published:** 2026-05-07

**Authors:** Saime Sundus Uygun, Busra Gokyer, Mehmet Ozturk, Evrim Kilicli, Osman Selcuk Duysak, Murat Konak

**Affiliations:** 1 Department of Neonatology, Faculty of Medicine, Selçuk University, Konya, Turkey; 2 Department of Pediatrics, Faculty of Medicine, Selçuk University, Konya, Turkey; 3 Department of Pediatric Radiology, Faculty of Medicine, Selçuk University, Konya, Turkey

**Keywords:** Neonatal nutrition, Oral feeding, Preterm infant, Suprahyoid muscles, Ultrasonography

## Abstract

The transition to full oral feeding (FOF) is a critical milestone for discharge in preterm infants and reflects the maturation of multiple physiological systems, particularly swallowing-related neuromuscular coordination. Suprahyoid muscles play a key role in swallowing by enabling elevation of the hyoid–laryngeal complex and protecting the airway. However, the relationship between the structural development of these muscles and the achievement of oral feeding in preterm infants remains insufficiently explored. This prospective single-center observational study included 28 preterm infants born at ≤ 32 weeks of gestation. Suprahyoid muscle thickness (right and left digastric, right and left mylohyoid, and geniohyoid muscles) was measured by ultrasonography at two time points: within the first 72 h of postnatal life and at the time of full oral feeding. Somatic growth parameters, including body weight, body length, and head circumference, were recorded simultaneously. Changes in muscle thickness over time, correlations with somatic growth parameters, and the predictive value of early muscle thickness for time to achieve FOF were analyzed. All suprahyoid muscle thicknesses increased significantly between the early postnatal period and the time of FOF (all *p* < 0.001). At the time of FOF, digastric muscle thickness ranged approximately between 2.7 and 3.2 mm, mylohyoid muscle thickness between 1.6 and 2.0 mm, and geniohyoid muscle thickness between 3.0 and 3.6 mm, with values appearing to cluster within a relatively narrow range during successful transition to oral feeding. A positive correlation was observed between body weight and certain muscle thickness measurements, particularly left mylohyoid (*r* = 0.453, *p* = 0.015) and right digastric muscles (*r* = 0.386, *p* = 0.043). However, suprahyoid muscle thickness measured within the first 72 h did not independently predict the time required to achieve FOF in multivariable regression analysis.

*Conclusion*: Suprahyoid muscle thickness increases significantly during the transition from early postnatal life to full oral feeding in preterm infants and appears to cluster within a relatively narrow range at the time of feeding maturation. Ultrasonographic evaluation of suprahyoid muscles may provide a non-invasive approach to describe structural changes associated with feeding maturation in preterm infants.

**Table:** 

**What is Known**: • *The transition to full oral feeding in preterm infants reflects the maturation of multiple physiological systems, including suck–swallow–breathing coordination, and is a key determinant of discharge readiness*. • *Ultrasonography has been used as a non-invasive method to assess structural and functional aspects of muscles involved in feeding and swallowing*.	
**What is New**: • *Suprahyoid muscle thickness in preterm infants increases significantly from early postnatal life to full oral feeding and appears to cluster within a relatively narrow range at the time of feeding maturation*. • *Early postnatal suprahyoid muscle thickness does not independently predict the time to achieve full oral feeding, suggesting that feeding maturation is a multifactorial process beyond a single structural parameter*.

**What is Known**:

• *The transition to full oral feeding in preterm infants reflects the maturation of multiple physiological systems, including suck–swallow–breathing coordination, and is a key determinant of discharge readiness*.

• *Ultrasonography has been used as a non-invasive method to assess structural and functional aspects of muscles involved in feeding and swallowing*.

**What is New**:

• *Suprahyoid muscle thickness in preterm infants increases significantly from early postnatal life to full oral feeding and appears to cluster within a relatively narrow range at the time of feeding maturation*.

• *Early postnatal suprahyoid muscle thickness does not independently predict the time to achieve full oral feeding, suggesting that feeding maturation is a multifactorial process beyond a single structural parameter*.

## Introduction

The World Health Organization defines preterm birth as birth occurring before the 37th week of pregnancy [1]. Preterm birth continues to be one of the leading causes of neonatal morbidity and mortality and remains a significant public health issue globally.

With advances in perinatal and neonatal care, the survival rates of premature infants have steadily increased. However, due to the structural and functional immaturity of their organ systems, premature infants may encounter numerous clinical complications [2]. In particular, the immaturity of the respiratory, cardiovascular, neurological, and gastrointestinal systems can lead to clinical problems such as susceptibility to infection, need for respiratory support, neurodevelopmental difficulties, and feeding problems, and these infants may require close monitoring in neonatal intensive care units [3].

Nutrition is one of the fundamental components of neonatal care for maintaining growth and development in premature infants [4]. However, the transition to oral feeding in premature infants is often delayed, which can prolong the length of hospital stay. Therefore, understanding the factors that influence the transition to oral feeding in premature infants is of great importance for clinical management [5]. Oral feeding in preterm infants is a complex process that depends not only on swallowing-related muscle function but also on the integration of multiple physiological components, including respiratory coordination, sucking ability, endurance, and behavioral state (vigilance). In clinical practice, feeding readiness is typically assessed using behavioral and physiological indicators such as sucking patterns, coordination, cardiorespiratory stability, and overall feeding tolerance.

Nevertheless, these assessments are largely based on clinical observation and may not fully capture the structural components underlying feeding development. In this context, ultrasonographic evaluation of muscle thickness may offer a non-invasive and objective approach to evaluating structural changes associated with feeding maturation. The primary objective of this study was to evaluate the thickness of suprahyoid muscles measured by ultrasonography during the transition to full oral feeding in premature infants. As a primary outcome, the thicknesses of the right and left digastric, right and left mylohyoid, and geniohyoid muscles were analyzed separately during full oral feeding (FOF). Additionally, the relationship between these muscle thicknesses and somatic growth parameters (body weight, body length, and head circumference) was investigated, and the potential value of muscle thickness measured in the early postnatal period in predicting the time required to achieve full oral feeding was evaluated.

## Materials and methods

### Study design and setting

This study was designed as a prospective, single-center observational study conducted in the Neonatal Intensive Care Unit of Selçuk University Faculty of Medicine Hospital. The study was conducted between June 2024 and January 2026 in the neonatal intensive care unit of a tertiary care center. In the preterm infants included in the study, suprahyoid muscle thicknesses were evaluated at two different time points in the same individuals. The first measurement was performed within the first 72 h of postnatal life, and the second measurement was obtained at the time when the infant achieved full oral feeding (FOF). Thus, the aim was to evaluate the change in suprahyoid muscle thickness between the early postnatal period and the time of full oral feeding in each infant.

### Participants

Preterm infants with a gestational age of ≤ 32 weeks and without major comorbidities other than prematurity were prospectively enrolled in this single-center observational study. Infants who were not intubated after birth or required intubation for ≤ 72 h and who achieved full oral feeding during follow-up were eligible for inclusion. Infants with genetic or chromosomal anomalies, central nervous system pathologies, major structural cardiac anomalies, surgical gastrointestinal diseases, gestational age > 32 weeks, or intubation longer than 72 h were excluded. A total of 28 preterm infants who completed follow-up were included in the final analysis. During the study period, a total of 36 preterm infants were assessed for eligibility. Of these, 28 met the inclusion criteria and were enrolled. Five infants were excluded due to predefined exclusion criteria, and three were not included due to incomplete data or failure to achieve full oral feeding.

### Clinical follow-up, measurements, and study variables

All infants were followed according to the standard feeding protocol of our unit. Enteral feeding was initially provided via an orogastric or nasogastric tube. In clinically stable infants, early buccal immunotherapy and/or trophic feeding was initiated. During follow-up, sucking-swallowing behavior, cardiorespiratory stability, and feeding tolerance were assessed, and infants were transitioned to cue-based feeding when appropriate before progressing to full oral feeding.

Full oral feeding (FOF) was defined as achieving an enteral intake of at least 150 mL/kg/day entirely by oral feeding, maintaining this intake for at least 48 h, and not requiring gavage support during this period. Continued weight gain, cardiorespiratory stability, and adequate urine and stool output were accepted as indicators of clinical stability. Time to achieve FOF was calculated by subtracting gestational age at birth from the postmenstrual age at FOF and was expressed in weeks.

Ultrasonographic assessments were performed at the bedside in the neonatal intensive care unit by a single pediatric radiologist with approximately 15 years of experience in pediatric ultrasonography, using a portable ultrasound device with a linear array transducer operating at 10 MHz. Infants were examined in the supine position with the head and neck in a neutral position. Measurements were obtained from long-axis images parallel to the muscle fibers, and muscle thickness was defined as the distance between the superior and inferior fascial borders using electronic calipers. Minimal probe pressure was applied, and measurements were obtained while infants were calm to minimize motion artifacts.

Suprahyoid muscle thickness was measured two times: within the first 72 h of postnatal life and at the time of FOF. The right and left digastric muscles, right and left mylohyoid muscles, and the geniohyoid muscle were evaluated at both time points.

At both measurement points, body weight, body length, and head circumference were recorded as indicators of somatic growth.

To improve standardization, all measurements were performed by the same observer using consistent anatomical landmarks. Ultrasonographic images were archived, and randomly selected images were re-measured by the same radiologist at a separate time point while blinded to the initial measurements to assess intra-observer consistency.

### Outcomes

The primary outcome was suprahyoid muscle thickness at the time of FOF, measured separately for the right and left digastric muscles, right and left mylohyoid muscles, and the geniohyoid muscle. Secondary outcomes included time to achieve FOF, change in muscle thickness between the first 72 h and FOF, and correlations between muscle thickness and somatic growth parameters. In addition, the predictive value of early postnatal muscle thickness for FOF was explored using linear regression analysis.

## Ethics approval

This study was approved by the Selçuk University Faculty of Medicine Local Ethics Committee (Decision No.: 2024/337, Meeting Date: 02.07.2024) and was conducted in accordance with the principles of the Declaration of Helsinki.

Written informed consent was obtained from the parents/legal guardians of all participants.

### Statistical analysis

All statistical analyses were performed using R version 4.1.2 (The R Foundation for Statistical Computing, Vienna, Austria). The distribution of continuous variables was assessed using the Shapiro–Wilk test and Q–Q plots. Normally distributed variables were presented as mean ± standard deviation, while non-normally distributed variables were expressed as median (minimum–maximum) or median (interquartile range), as appropriate.

Suprahyoid muscle thickness at the time of full oral feeding (FOF), the primary outcome of the study, was analyzed separately for each muscle group using descriptive statistics. Changes in muscle thickness between the first 72 h of postnatal life and the time of FOF, as well as changes in somatic growth parameters (body weight, body length, and head circumference), were evaluated using paired-samples *t*-tests or Wilcoxon signed-rank tests, as appropriate.

Time to achieve FOF was calculated by subtracting gestational age at birth from the postmenstrual age at FOF and was expressed in weeks. Associations between muscle thickness and somatic growth parameters, as well as time to achieve FOF, were assessed using Pearson or Spearman correlation analyses, depending on data distribution.

The potential predictive value of suprahyoid muscle thickness measured within the first 72 h of postnatal life for time to achieve FOF was evaluated using linear regression analysis. Both univariate and multivariable models were constructed, with adjustment for gestational age at birth and birth weight as potential confounders.

Regression results were reported as *β* coefficients with 95% confidence intervals. A *p*-value < 0.05 was considered statistically significant.

## Results

A total of 28 preterm infants were included in the study, of whom 57.1% (*n* = 16) were male. Baseline characteristics of the study population are presented in Table 1.

**Table 1:** Baseline characteristics of the study population (*n* = 28)

Variable	Value
Gestational age at birth (weeks)	30.54 ± 1.79
Birth weight (g)	1486.79 ± 458.00
Growth status, *n* (%)	AGA: 26 (92.9) SGA: 1 (3.6) LGA: 1 (3.6)
Mode of delivery, *n* (%)	C/S: 25 (89.3), Vaginal: 3 (10.7)
Sex, *n* (%)	Male 16 (57.1) Female 12 (42.9)
5-min Apgar score, median (min–max)	8 [5–9]
Postnatal age at first measurement (days)	≤ 3 days
Postmenstrual age at FOF (weeks)	36.12 ± 1.76
Time to achieve FOF (weeks)	5.61 ± 2.63

*SGA* small for gestational age, *AGA* appropriate for gestational age, *C/S* cesarean section

Continuous variables are presented as mean ± standard deviation or median (minimum–maximum), as appropriate

*Time to achieve FOF = postmenstrual age at FOF − gestational age at birth (weeks)

The first ultrasonographic assessment was performed within the first 72 h of postnatal life, and the second was performed at the time of full oral feeding. The mean postmenstrual age at FOF was 36.12 ± 1.76 weeks, and the mean time to achieve FOF was 5.61 ± 2.63 weeks.

Body weight, body length, and head circumference increased significantly between the two measurement time points (all *p* < 0.001). These changes are presented in Table 2.

**Table 2:** Changes in somatic growth parameters between the first 72 h and full oral feeding (FOF)

Variable	First 72 h	FOF period	*p*
Body weight (g)	1486.79 ± 458.00	2240.36 ± 421.48	< 0.001
Body length (cm)	39.18 ± 3.71	45.04 ± 3.44	< 0.001
Head circumference (cm)	28.48 ± 2.76	32.00 ± 1.41	< 0.001

Data are presented as mean ± standard deviation. Comparisons were performed using paired *t*-test

All suprahyoid muscle thickness measurements increased significantly between the early postnatal period and the time of FOF (all *p* < 0.001). Detailed comparisons of the right and left digastric muscles, right and left mylohyoid muscles, and geniohyoid muscle are presented in Table 3. Detailed data regarding the comparison of muscle thickness between the two points are presented in Table 3.

**Table 3:** Comparison of suprahyoid muscle thickness between the first 72 h of postnatal life and the full oral feeding (FOF) period (*n* = 28)

Muscle	First 72 h	FOF period	Δ (FOF – first 72 h)	*p*
Right digastric	2.10 (1.80–2.40)	2.95 (2.68–3.23)	0.80 (0.50–1.00)	< 0.001
Left digastric	2.20 (1.90–2.40)	3.00 (2.80–3.23)	0.75 (0.58–0.93)	< 0.001
Right mylohyoid	1.19 ± 0.39	1.80 (1.58–1.95)	0.61 ± 0.40	< 0.001
Left mylohyoid	1.16 ± 0.36	1.80 (1.60–2.03)	0.64 ± 0.42	< 0.001
Geniohyoid	2.43 ± 0.64	3.20 (2.98–3.60)	0.77 ± 0.64	< 0.001

Continuous variables are presented as mean ± standard deviation or median (25th–75th percentile) according to distribution. Comparisons were performed using the paired t-test or the Wilcoxon signed-rank test

According to the data presented in Table 3, suprahyoid muscle thicknesses were observed to cluster within a specific range during the full oral feeding (FOF) period. At the time of FOF, digastric muscle thickness ranged approximately between 2.7 and 3.2 mm, mylohyoid muscle thickness between 1.6 and 2.0 mm, and geniohyoid muscle thickness between 3.0 and 3.6 mm. At the time of FOF, muscle thickness values were observed to cluster within a relatively narrow range, which may descriptively reflect structural maturation during the transition to oral feeding in this cohort.

The relationships between suprahyoid muscle thickness measured during the full oral feeding (FOF) period and somatic growth parameters were evaluated using correlation analysis.

Correlation analyses showed a positive association between body weight at FOF and selected muscle thickness measurements. A statistically significant correlation was observed for left mylohyoid muscle thickness (*r* = 0.453, *p* = 0.015) and right digastric muscle thickness (*r* = 0.386, *p* = 0.043), whereas no significant associations were found for body length or head circumference (Table 4).

**Table 4:** Correlation analysis between suprahyoid muscle thickness and somatic growth parameters during the full oral feeding (FOF) period (*n* = 28)

Muscle	Body weight (*r*, *p*)	Body length (*r*, *p*)	Head circumference (*r*, *p*)
Right digastric	0.386; 0.043	0.21; 0.28	0.18; 0.34
Left digastric	0.373; 0.051	0.19; 0.33	0.16; 0.41
Right mylohyoid	0.372; 0.051	0.17; 0.38	0.14; 0.47
Left mylohyoid	0.453; 0.015	0.22; 0.27	0.19; 0.31
Geniohyoid	0.203; 0.30	0.15; 0.44	0.12; 0.52

Correlation analyses were performed using Pearson or Spearman methods according to the distribution of the variables. *R*, Pearson correlation coefficient; *ρ*, Spearman correlation coefficient. A *p*-value < 0.05 was considered statistically significant

Additionally, no significant association was found between the change in suprahyoid muscle thickness (Δ muscle thickness) and the changes in somatic growth parameters (all *p* > 0.05). Detailed results of the correlation analysis are presented in Table 4.

The effect of suprahyoid muscle thickness measured within the first 72 h of postnatal life on the time required to achieve full oral feeding (FOF) was evaluated using linear regression analysis. In regression analyses, suprahyoid muscle thickness measured within the first 72 h of postnatal life was not independently associated with time to achieve FOF after adjustment for gestational age at birth and birth weight (Table 5).

**Table 5:** Effect of suprahyoid muscle thickness measured within the first 72 h of postnatal life on time to achieve full oral feeding: linear regression analysis (*n* = 28)

Variable (72 h)	Univariate *β* (95% CI)	*p*	Multivariable β* (95% CI)	*p*
Right digastric	− 0.88 (− 2.25 to 0.49)	0.20	− 0.64 (− 2.41 to 1.13)	0.46
Left digastric	− 0.91 (− 2.36 to 0.54)	0.21	− 0.71 (− 2.56 to 1.14)	0.44
Right mylohyoid	− 0.73 (− 2.02 to 0.56)	0.25	− 0.58 (− 2.31 to 1.15)	0.50
Left mylohyoid	− 0.79 (− 2.15 to 0.57)	0.24	− 0.63 (− 2.42 to 1.16)	0.47
Geniohyoid	− 0.82 (− 2.30 to 0.66)	0.27	− 0.53 (− 2.25 to 1.19)	0.35

*Multivariable model adjusted for gestational age at birth and birth weight

The *β* coefficients and 95% confidence intervals obtained from the regression analysis are presented in Table 5.

## Discussion

The transition to full oral feeding in premature infants is one of the most important milestones determining discharge from neonatal intensive care [6]. Readiness for oral feeding is generally considered an indicator of neurological maturation and is particularly related to the adequate development of sucking–swallowing–breathing coordination. In addition to swallow-related coordination, other factors such as endurance, vigilance, and the ability to maintain stable breathing during feeding are essential contributors to successful oral feeding. The literature reports that this coordination matures in most preterm infants at approximately 34–36 weeks postmenstrual age [7]. However, clinical observations show that the timing of transition to full oral feeding can vary significantly among preterm infants of similar postmenstrual age. This observation suggests that the transition to oral feeding may not be fully explained by neurological maturation alone. Previous studies have shown that oral feeding skills develop with increasing postmenstrual age, particularly in very low birth weight preterm infants, through functional changes such as increased sucking pressure and more regular swallowing frequency [8]. In addition, the structural development of muscle groups involved in somatic growth and feeding may also play an important role in this process [9].

In our study, the increased suprahyoid muscle thickness observed on ultrasonography may reflect structural changes associated with functional maturation [10]. Taken together, these findings should therefore be interpreted as descriptive observations within this specific cohort rather than as evidence of a causal relationship.

Ultrasonography is a safe imaging method that allows non-invasive, repeatable, and bedside assessment of the muscle groups involved in the swallowing process [11, 12]. Its ability to avoid radiation exposure in premature infants and to perform repeated measurements provides significant advantages for clinical use [13].

In recent years, studies using ultrasonography to examine muscle structure for the assessment of oral feeding maturity have increased. Nagel and colleagues used ultrasonography to evaluate abdominal muscle thickness in preterm infants and reported that greater muscle thickness may be associated with achieving full oral feeding at an earlier postmenstrual age [14]. Similarly, Capilouto and colleagues measured the thickness of the posterior tongue using ultrasound during feeding and non-feeding sucking in term and preterm infants and showed that tongue thickness was related to sucking strength [15]. Furthermore, Geddes and his colleagues used ultrasound to visualize the swallowing mechanisms of breastfed full-term infants and showed that this method allows for a non-invasive assessment of swallowing function [16].

Furthermore, several studies have reported that measuring muscle thickness from a single cross-section shows a strong correlation with total muscle volume and can reliably reflect muscle hypertrophy [17]. Taking together, these findings indicate that ultrasonography is a reliable and practical method for evaluating the structural characteristics of muscle groups involved in oral feeding. In the present study, evaluating the suprahyoid muscle group, which plays a critical role in the swallowing mechanism, rather than the tongue or abdominal muscles, provides a different perspective to the existing literature.

The suprahyoid muscle group (digastric, mylohyoid, and geniohyoid muscles) plays a fundamental role in protecting the airway during swallowing by enabling the anterosuperior elevation of the hyoid and hyolaryngeal complex and contributes to the opening of the upper esophageal sphincter. Therefore, the structural integrity and development of this muscle group is considered important for safe and effective swallowing function [18]. However, studies objectively evaluating the relationship between suprahyoid muscle development and the transition to oral feeding remain limited [19].

Although the present study focused on the suprahyoid muscle group, tongue muscles also play a crucial role in feeding and swallowing function, particularly in generating suction and coordinating bolus transport. The absence of tongue muscle assessment in this study limits the ability to fully evaluate all structural contributors to feeding development. Future studies incorporating both suprahyoid and tongue muscle measurements may provide a more comprehensive understanding of the structural basis of feeding maturation in preterm infants.

In the present study, suprahyoid muscle thickness increased significantly from the early postnatal period to the full oral feeding (FOF) in preterm infants. In addition, suprahyoid muscle thickness values at the time of FOF were observed to cluster within a specific range. Digastric muscle thickness ranged approximately between 2.7 and 3.2 mm, mylohyoid muscle thickness between 1.6 and 2.0 mm, and geniohyoid muscle thickness between 3.0 and 3.6 mm. At the time of full oral feeding, muscle thickness values appeared to cluster within a relatively narrow range in this cohort. As also emphasized by Şahan et al., this finding suggests that the transition to oral feeding may reflect a physiological maturation process associated with the development of oral motor structures involved in feeding and swallowing [20].

In our study, the relationship between suprahyoid muscle thickness measured at the time of full oral feeding (FOF) and somatic growth parameters was also evaluated. A positive correlation was observed particularly between body weight and certain suprahyoid muscle thickness measurements. Although statistically significant, the strength of the observed correlations was modest and should be interpreted with caution. This finding suggests that the increase in muscle thickness may reflect a component of overall somatic growth. Considering that feeding function in the neonatal period is a dynamic process associated with the maturation of multiple physiological systems, the development of oral motor structures and swallowing-related muscle groups may progress in parallel with general growth processes [21]. However, no significant correlation was observed between muscle thickness and body length or head circumference. This finding suggests an association between feeding-related muscle development and certain somatic growth indicators, such as body weight. These findings should be interpreted cautiously given the modest strength of the observed correlations.

In our study, suprahyoid muscle thickness measured within the first 72 h of postnatal life did not independently predict the time required to achieve full oral feeding. This finding suggests that the transition to oral feeding cannot be explained by a single morphological parameter. Instead, it appears to represent a multidimensional developmental process determined by the interaction of several factors, including neurological maturation, somatic growth, and functional coordination [22]. In preterm infants, successful oral feeding depends on the interaction of multiple physiological systems, including neurological maturation, cardiorespiratory stability, muscle strength, and suck–swallow coordination. This finding highlights that early structural measurements alone may be insufficient to predict feeding outcomes and is consistent with the concept that oral feeding maturation in preterm infants is a multifactorial process.

This study has several important limitations. First, it was conducted in a single-center with a relatively small sample size, which limits the generalizability of the findings and reduces statistical power. Second, only infants who achieved full oral feeding were included in the analysis, which may introduce selection bias. Third, although multivariable regression analysis was performed, the ability to adjust for potential confounders was limited, and important clinical variables, such as comorbidities and feeding-related factors, may not have been fully accounted for.

In addition, all ultrasonographic measurements were performed by a single observer, and inter-observer variability could not be assessed. Quantitative measures of intra-observer agreement (e.g., intraclass correlation coefficient) and the number of re-evaluated images were not recorded, which limits the assessment of measurement reliability.

Furthermore, important functional aspects of feeding, such as endurance, behavioral state (vigilance), and respiratory coordination, as well as tongue muscle structure, were not evaluated in this study, limiting the ability to fully interpret the role of muscle thickness within the complex process of feeding development.

Finally, the absence of a comparison group and the observational design of the study limit the interpretability of the findings and preclude causal inference.

## Conclusion

In conclusion, this study showed that suprahyoid muscle thickness in preterm infants increased significantly from the early postnatal period to the time of full oral feeding. At the time of full oral feeding, muscle thickness values appeared to cluster within a relatively narrow range in this cohort.

These findings suggest that the transition to oral feeding may not be fully explained by neurological maturation alone and may also involve structural changes in muscle groups associated with feeding and swallowing. However, early measurements of suprahyoid muscle thickness did not independently predict the time to achieve full oral feeding, indicating that feeding maturation is likely a multifactorial process. These findings are consistent with the concept that feeding maturation is influenced by multiple interacting components, including respiratory stability, endurance, behavioral state, and suck–swallow–breathe coordination, rather than a single structural parameter.

Ultrasonographic evaluation of the suprahyoid muscle group may provide a non-invasive method to describe structural changes during the transition to oral feeding; however, its clinical utility as a predictive tool remains uncertain. Future multicenter studies with larger sample sizes are needed to further clarify these relationships and to determine the potential clinical applicability of these findings.

## Data Availability

The datasets generated and/or analyzed during the current study are not publicly available due to patient confidentiality but are available from the corresponding author on reasonable request. The authors declare no competing interests.
